# IL-27 amplifies cytokine responses to Gram-negative bacterial products and *Salmonella typhimurium* infection

**DOI:** 10.1038/s41598-018-32007-y

**Published:** 2018-09-12

**Authors:** C. Petes, N. Odoardi, S. M. Plater, N. L. Martin, K. Gee

**Affiliations:** Department of Biomedical and Molecular Sciences, Queen’s University, Kingston, ON K7L 3N6 Canada

## Abstract

Cytokine responses from monocytes and macrophages exposed to bacteria are of particular importance in innate immunity. Focusing on the impact of the immunoregulatory cytokine interleukin (IL)-27 on control of innate immune system responses, we examined human immune responses to bacterial products and bacterial infection by *E. coli* and *S. typhimurium*. Since the effect of IL-27 treatment in human myeloid cells infected with bacteria is understudied, we treated human monocytes and macrophages with IL-27 and either LPS, flagellin, or bacteria, to investigate the effect on inflammatory signaling and cytokine responses. We determined that simultaneous stimulation with IL-27 and LPS derived from *E. coli* or *S. typhimurium* resulted in enhanced IL-12p40, TNF-α, and IL-6 expression compared to that by LPS alone. To elucidate if IL-27 manipulated the cellular response to infection with bacteria, we infected IL-27 treated human macrophages with *S. typhimurium*. While IL-27 did not affect susceptibility to *S. typhimurium* infection or *S. typhimurium*-induced cell death, IL-27 significantly enhanced proinflammatory cytokine production in infected cells. Taken together, we highlight a role for IL-27 in modulating innate immune responses to bacterial infection.

## Introduction

The cytokine interleukin (IL)-27, first described by Pflanz *et al*. in 2002, is part of the IL-12 family of heterodimeric cytokines which have fundamental roles in innate and adaptive immune regulation^1,2^. IL-27, composed of IL-27p28 (p28) and Epstein Barr Virus-induced gene 3 (EBI3) subunits, is produced in response to bacterial infection in myeloid cells and exhibits both pro- and anti-inflammatory functions^2,3^. The receptor complex bound by IL-27 is comprised of IL-27Rα (WSX-1) and glycoprotein (gp)-130^4^. The ubiquitously expressed gp130 receptor subunit is shared among all cytokines belonging to the IL-6 family, resulting in signaling capability across a variety of immune and non-immune cells^5^. In comparison, WSX-1 is primarily expressed on lymphocytes, including naïve T cells, monocytes, and dendritic cells^4^. Primary human monocytes and activated macrophages express WSX-1 and gp130, and therefore possess the ability to respond to IL-27 stimulation^4,6^.

In adaptive immune responses, IL-27 is well-characterized to promote differentiation to type I helper T cell (Th1) lineages for interferon (IFN)-γ production by CD4+ T cells^2,7^; however, recent reports document that IL-27 also affects monocytes and macrophages^8–13^. Specifically, IL-27 enhances major histocompatibility complex (MHC) class I and II expression and modulates inflammatory cytokine production from monocytes and macrophages^4,9,10^. Furthermore, monocytes exposed to IL-27 have augmented Toll-like receptor (TLR)-4 expression and enhanced lipopolysaccharide (LPS)-induced inflammatory cytokine production^11,13^. These findings support a role for IL-27 in anti-microbial defenses by altering expression of innate immune sensors such as TLR4.

TLRs are pattern recognition receptors that detect viral and bacterial components and initiate immune responses required for clearance of infection^14–16^. Pathogen-associated molecular patterns such as LPS and flagellin are recognized by TLR4 and TLR5, respectively^17,18^; TLR-mediated signaling responses are essential in the protection against Gram-negative bacterial infections such as *Escherichia coli* and *Salmonella typhimurium*^14–16^. Direct LPS stimulation of macrophages induces significant changes in gene expression, signaling, and cytokine induction comparable to *S. typhimurium* infection, indicating that LPS stimulation alone can model macrophage responses to Gram-negative bacteria^19–21^. Signaling via TLR4 or TLR5 initiates the myeloid differentiation primary response gene 88 (MyD88)-dependent cascade and nuclear factor (NF)-κB-mediated production of proinflammatory cytokines, including tumor necrosis factor (TNF)-α, IL-6, and IL-12p40^18,22,23^.

Based on findings that IL-27 upregulates TLR4 expression in monocytes^11^, this study investigates whether IL-27 can modulate macrophage responses to bacterial products including LPS and flagellin, or infection with live bacteria. Our data demonstrate that IL-27 enhances TLR4 and TLR5 expression, potentiating greater NF-κB/activator protein (AP)-1 signaling by monocytes in response to LPS and flagellin stimulation respectively. Although IL-27 had no effects on *S. typhimurium* cellular invasion or bacterial-induced cell death, IL-27 pre-treatment of macrophages followed by stimulation with LPS derived from *S. typhimurium* or infection with *S. typhimurium* resulted in amplified proinflammatory cytokine production compared to untreated cells. Taken together, our data highlight a novel role for IL-27 in increasing TLR4 and TLR5 expression in human monocytes and macrophages, in addition to immunomodulatory functions on proinflammatory cytokine production in response to Gram-negative bacterial products or *S. typhimurium* infection.

## Results

### Co-stimulation of LPS and IL-27 upregulates proinflammatory cytokine expression in myeloid cells

**Figure 1:**
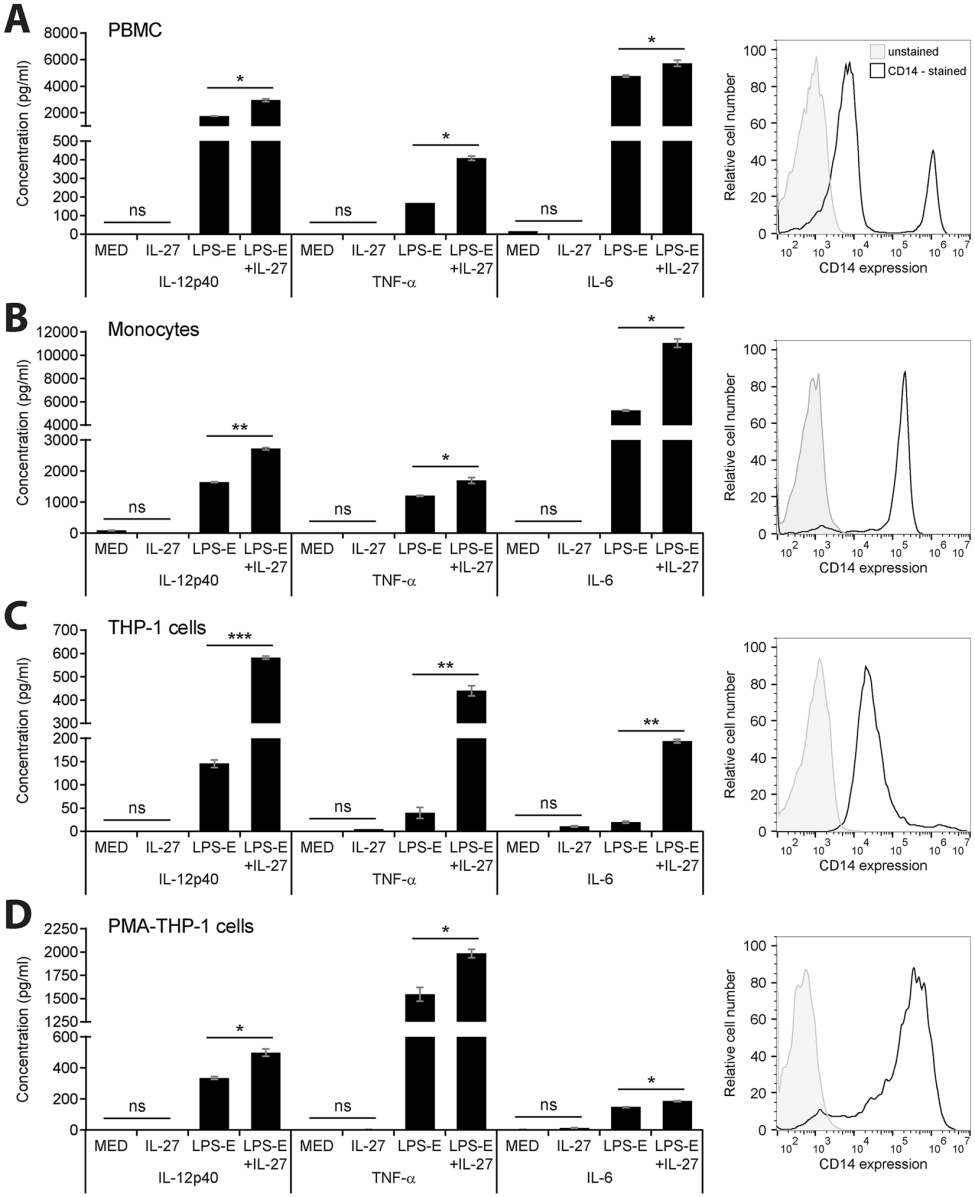
Co-stimulation with IL-27 enhanced LPS-induced cytokine production in human myeloid cells. Human PBMC (**A**), primary human monocytes (**B**), THP-1 cells (**C**), and PMA-THP-1 cells (**D**) were stimulated with LPS-E (100 ng/ml), IL-27 (50 ng/ml), or both LPS-E plus IL-27 concomitantly for 24 hours. IL-12p40, TNF-α and IL-6 levels (*left panels*) were measured in cell-free supernatants by ELISA. CD14 expression was measured by flow cytometry in resting cells (*right panels)*. Histograms include CD14 expression and autofluorescence of each cell type (A–D, *right panels*). Data are representative of at least three different blood donors or independent replicate experiments showing mean and standard deviation of ELISA technical replicates from one experiment. Mann Whitney U tests were used for statistical analyses between pairs as indicated. ns = not significant; *p≤0.05; **p≤0.01; ***p≤0.001.

Previous studies have demonstrated that pre-treatment with IL-27 enhances *E. coli* LPS responsiveness and cytokine production in human immune cells via TLR4 upregulation, while co-treatment with LPS and IL-27 enhances inflammasome activity and IL-1β expression^11,13^. We initially tested responses of human peripheral blood mononuclear cells (PBMC) and primary human monocytes as well as those of the human monocytic cell line, THP-1. To model how IL-27 stimulation affects monocytes versus macrophages, we compared responses of THP-1 cells and PMA-differentiated THP-1 cells (PMA-THP-1). All cells were stimulated with *E. coli* LPS (LPS-E; 100 ng/ml) and recombinant human IL-27 (50 ng/ml) overnight. Secreted levels of IL-12p40, TNF-α, and IL-6 were quantified in cell-free supernatants by ELISA. Each cell type produced all cytokines examined in response to either LPS-E alone or IL-27 plus LPS-E (Fig. 1A–D). Furthermore, IL-27 alone did not induce detectable cytokine production, but simultaneous addition of IL-27 and LPS significantly enhanced IL-12p40, TNF-α, and IL-6 production in all cells. In comparison to THP-1 cells, PMA-THP-1 cells exhibited higher levels of cytokine induction after 24 hours in response to LPS alone, whereas THP-1 cells exhibited a greater increase upon IL-27 co-stimulation (Fig. 1C,D). As expected, levels of CD14 expression correlated with LPS-responsiveness (Fig. 1, *right panel*) as CD14 is a co-receptor for TLR4-mediated LPS recognition^24,25^. Since PBMC are a mixed population of cells including monocytes, lymphocytes and granulocytes, CD14-high cells only comprise 10% of the population, and therefore these cells exhibit less LPS and IL-27/LPS-induced cytokine expression compared to purified monocytes (Fig. 1A,B, *right panels*). Similarly, PMA-THP-1 cells exhibit higher CD14 expression compared to THP-1 cells, correlating with relatively higher LPS-responses in PMA-THP-1 cells compared to THP-1 cells (Fig. 1C,D, *right panels*). Taken together, these results indicate that IL-27 enhances LPS responsiveness, although to a lesser extent in macrophages compared to monocytes. To further analyze the differential responsiveness observed in monocytes versus macrophages, we focused the subsequent experiments on THP-1 and PMA-THP-1 cells.

### IL-27 enhances inducible NF-κB/AP-1 signaling in THP-1 cells, but not PMA-THP-1 cells

**Figure 2:**
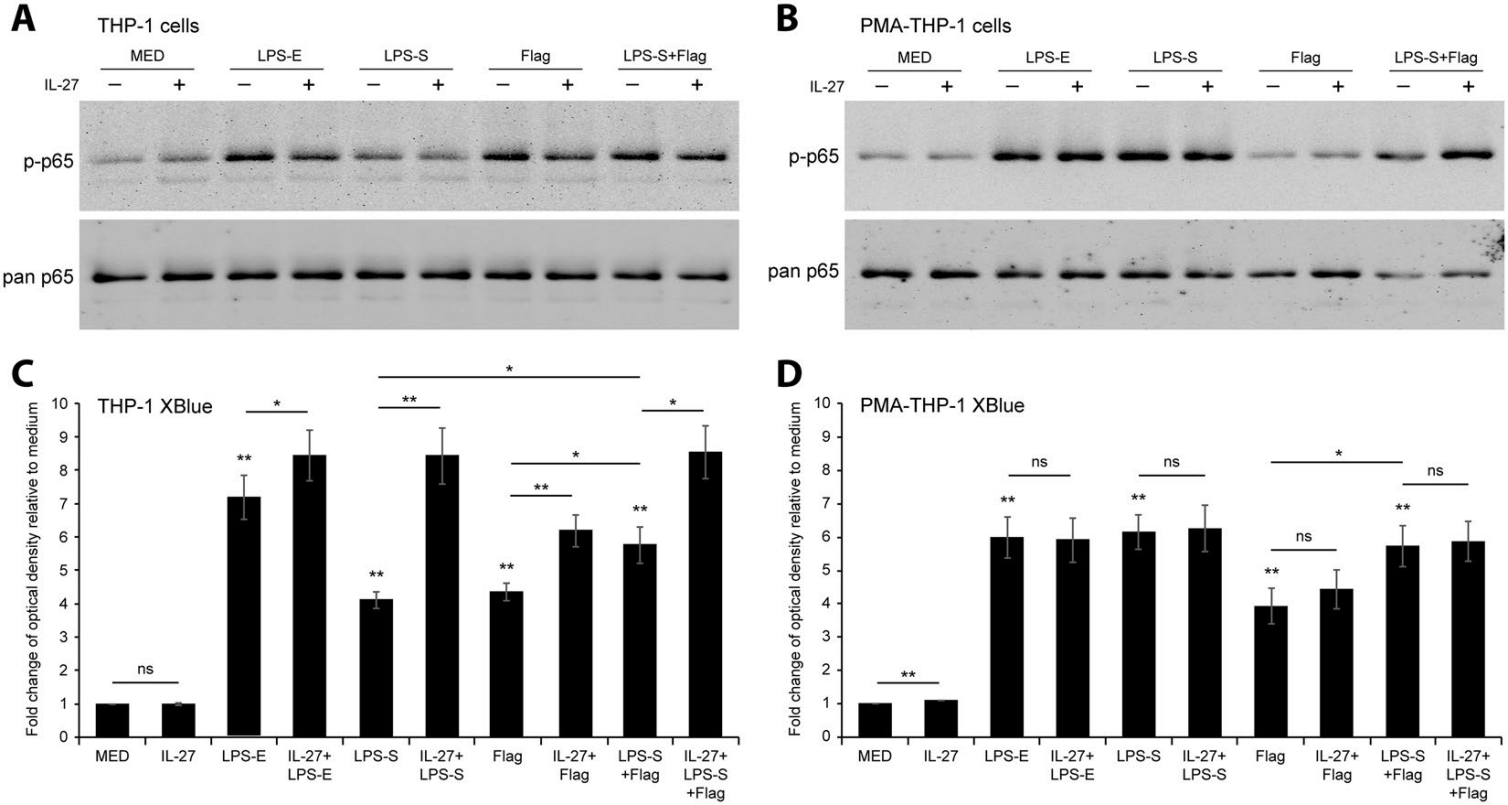
IL-27 altered TLR-mediated NF-κB/AP-1 activity in THP-1 monocytes, but not in PMA-THP-1 macrophages. THP-1 cells (**A**) and PMA-THP-1 cells (**B**) were stimulated with LPS-E (100 ng/ml), LPS-S (100 ng/ml), flagellin (Flag) (500 ng/ml), IL-27 (50 ng/ml), or combinations of TLR agonists plus IL-27 concomitantly as indicated for 15 minutes. Phosphorylation of NF-κBp65 subunit (p-p65) was presented using immunoblotting on whole cell lysates. Membranes were stripped and re-probed for pan NF-κBp65 (pan p65) as a loading control. Images shown are representative of three independent experiments. THP-1 XBlue cells (**C**) and PMA-THP-1 XBlue cells (**D**) were stimulated with LPS-E (100 ng/ml), LPS-S (100 ng/ml), flagellin (Flag) (500 ng/ml), IL-27 (50 ng/ml), or combinations of TLR agonists plus IL-27 concomitantly as indicated for 24 hours to allow for NF-κB/AP-1-induced SEAP production and secretion. SEAP production was quantified using a colorimetric QUANTI-Blue™ assay. Fold changes relative to medium controls (MED) were calculated. Data represent the mean and standard deviation of triplicate experiments. Mann Whitney U tests were used for statistical analyses between TLR agonists compared to MED (no lines) or between pairs as indicated (lines). ns = not significant; *p≤0.05; **p≤0.01.

Activation of the NF-κB and AP-1 transcription factors is key to LPS-induced cytokine expression^26^; furthermore, we have previously demonstrated that IL-27-mediated cytokine production is dependent on NF-κB activity^10^. Thus, to assess the effect of co-delivery of IL-27 and LPS on TLR4-mediated signaling, we measured NF-κBp65 phosphorylation by immunoblot in THP-1 and PMA-THP-1 cells co-stimulated with *E. coli*-derived LPS (LPS-E) or *S. typhimurium*-derived LPS (LPS-S) at 100 ng/ml or flagellin at 500 ng/ml in the presence or absence of IL-27 (50 ng/ml) for 15 minutes. In the absence of IL-27, both cell types exhibited NF-κBp65 phosphorylation in response to LPS-E, LPS-S, and LPS-S + Flag as expected (Fig. 2A,B). In THP-1 cells, flagellin induced a greater induction of NF-κBp65 phosphorylation compared to PMA-THP-1 cells. IL-27 stimulation alone slightly enhanced NF-κBp65 phosphorylation relative to medium control in THP-1 cells; however, co-stimulation with IL-27 plus TLR4 and TLR5 agonists resulted in decreased NF-κBp65 phosphorylation in these cells (Fig. 2A). In contrast, IL-27 co-treatment of PMA-THP-1 cells negligibly altered LPS-E-, LPS-S-, or flagellin-mediated NF-κBp65 phosphorylation relative to total NF-κBp65 levels (Fig. 2B).

In addition to examining phosphorylation of NF-κBp65, we examined the combined effect of NF-κB and AP-1 activation using THP-1 XBlue cells. These cells express an NF-κB/AP-1-inducible, secreted embryonic alkaline phosphatase (SEAP) reporter gene and provide a robust and quantitative readout of transcriptional activity^27–30^. THP-1 XBlue and PMA-THP-1 XBlue cells were co-stimulated with LPS-E or LPS-S at 100 ng/ml or flagellin at 500 ng/ml in the presence or absence of IL-27 (50 ng/ml) overnight. In THP-1 XBlue cells, IL-27 alone did not significantly activate NF-κB/AP-1 (Fig. 2C), in contrast to our previous study^11^; this discrepancy is due to the use of a lower dose of IL-27 (50 ng/ml compared to 100 ng/ml) in the current study. Dose response experiments in THP-1 XBlue cells and PMA-THP-1 XBlue cells revealed that stimulation with IL-27 at 100 ng/ml yielded significantly enhanced NF-κB/AP-1 activity relative to 50 ng/ml of IL-27 and unstimulated cells (data not shown). Furthermore, in PMA-THP-1 XBlue cells, IL-27 (50 ng/ml) induced NF-κB/AP-1 activity, though this was a moderate response relative to TLR agonists (Fig. 2D). In THP-1 XBlue cells, both LPS-E and LPS-S induced NF-κB/AP-1 activation, which was significantly enhanced by simultaneous IL-27 treatment (Fig. 2C), in contrast to levels of NF-κB phosphorylation (Fig. 2A). In PMA-THP-1 XBlue cells, NF-κB/AP-1 activity was comparable between LPS-E, LPS-S, or LPS-S + Flag with or without the addition of IL-27 (Fig. 2D) similar to phosphorylation of NF-κBp65 (Fig. 2B). Taken together, these results indicate a differential response to IL-27 treatment between monocytes and macrophages and suggest the involvement of a combination of NF-κB and AP-1 transcription factor activation.

To further investigate the effect of IL-27 on cytokine responses initiated by bacterial agonists, we compared responses from LPS-E and LPS-S. In both THP-1 and PMA-THP-1 XBlue cells, LPS-E and LPS-S stimulation resulted in significant NF-κB/AP-1 activation compared to unstimulated controls (Fig. 2C,D). In THP-1 XBlue cells, LPS-E induced greater NF-κB/AP-1 activation compared to LPS-S (Fig. 2C), while in PMA-THP-1 XBlue cells both LPS-E and LPS-S demonstrated comparable NF-κB/AP-1 activation (Fig. 2D). Treatment with IL-27 resulted in significantly higher LPS-induced NF-κB/AP-1 activity in THP-1 XBlue cells, but not in PMA-THP-1 XBlue cells. Additionally, IL-27 co-stimulation with LPS-S resulted in a greater fold change in NF-κB/AP-1 activity than with LPS-E in THP-1 XBlue cells (Fig. 2C). Interestingly, stimulation with *S. typhimurium* flagellin alone resulted in enhanced NF-κB/AP-1 activity and the addition of IL-27 resulted in a moderate upregulation of flagellin-induced NF-κB/AP-1 activity in both cell types, although this did not reach statistical significance in PMA-THP-1 cells (Fig. 2C,D). Addition of LPS-S and flagellin together resulted in a greater NF-κB/AP-1 activity compared to either LPS-S or flagellin alone in THP-1 XBlue cells, and this was further increased with IL-27 co-treatment. However, in PMA-THP-1 XBlue cells, addition of LPS-S and flagellin did not increase NF-κB/AP-1 activity over that of LPS-S alone and addition of IL-27 did not enhance NF-κB/AP-1 activity under these conditions. Taken together, IL-27 enhances LPS signaling capacity in THP-1 cells but not in PMA-THP-1 cells. Moreover, IL-27 enhanced responses to LPS-S to a greater extent than that for LPS-E in THP-1 cells. Thus, in subsequent experiments we focused on examining the effects of IL-27 on the response to *S. typhimurium* components and infection.

### TLR4 and TLR5 expression are enhanced by IL-27

**Figure 3:**
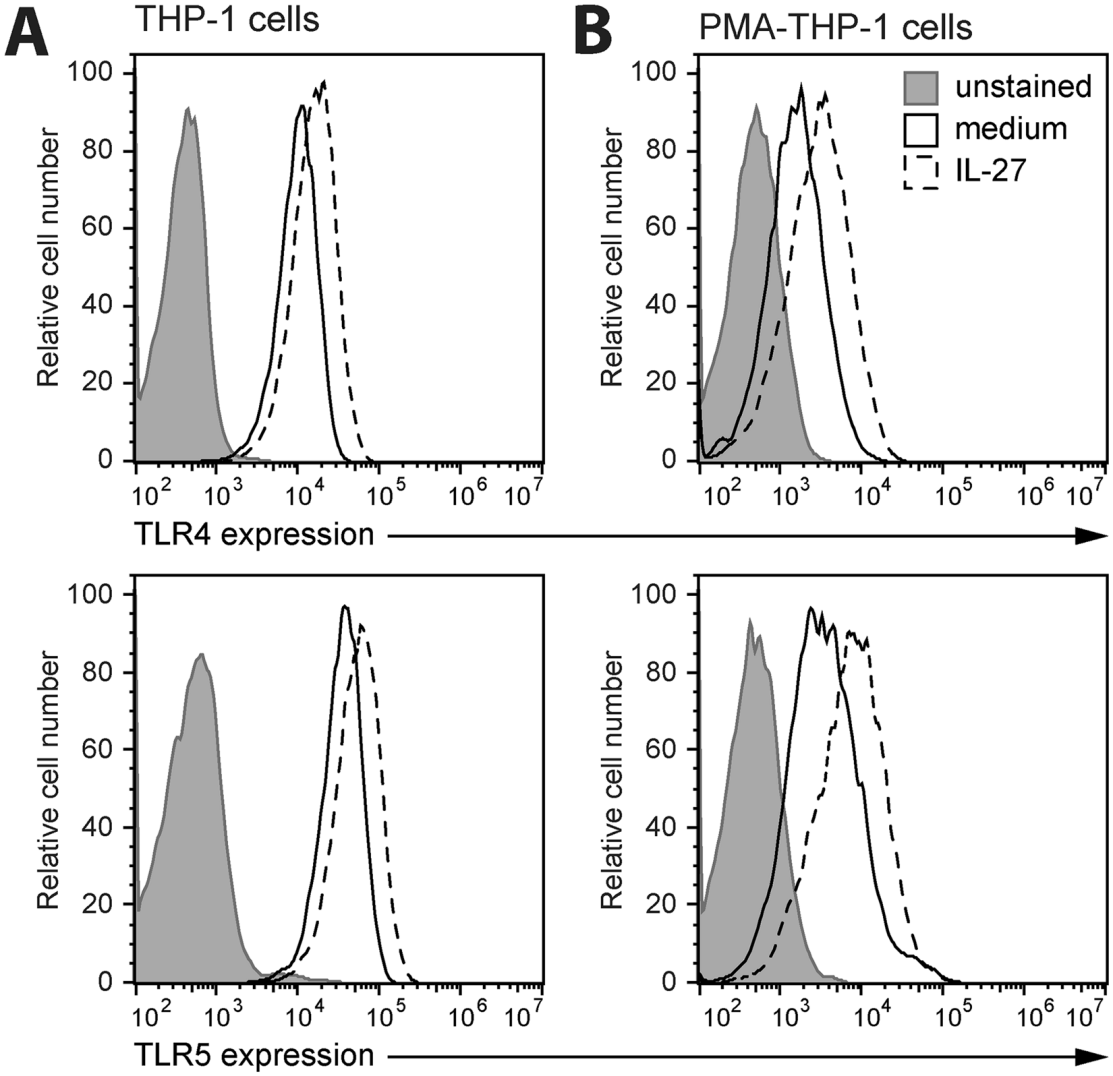
Stimulation with IL-27 increased TLR4 and TLR5 expression in monocytes and macrophages. THP-1 cells (**A**) and PMA-THP-1 cells (**B**) were stimulated with or without IL-27 (50 ng/ml) for 16 hours. Cells were stained with anti-human TLR4 (*top panels*) or TLR5 (*bottom panels*) antibodies for receptor expression quantification by flow cytometry. Unstained cells were acquired to quantify autofluorescence of each cell type. Data are representative of at least three independent replicate experiments.

To determine if the effects of IL-27 on NF-κB/AP-1 activation in response to *S. typhimurium* components were due to different levels of TLR4 and TLR5 expression, we treated THP-1 and PMA-THP-1 cells with or without IL-27 (50 ng/ml) for 16 hours and measured surface expression of TLR4 and TLR5 by flow cytometry. Interestingly, basal levels of both TLR4 and TLR5 expression were higher in THP-1 cells compared to PMA-THP-1 cells (Fig. 3A,B). Moreover, TLR4 and TLR5 expression levels were enhanced in response to IL-27 treatment compared to unstimulated controls in both THP-1 and PMA-THP-1 cells (Fig. 3A,B). This suggests that IL-27 may enhance responses to bacterial components by upregulating TLR4 and TLR5 expression in monocytes and macrophages.

### IL-27 enhances cytokine responses to *S. typhimurium* components

**Figure 4:**
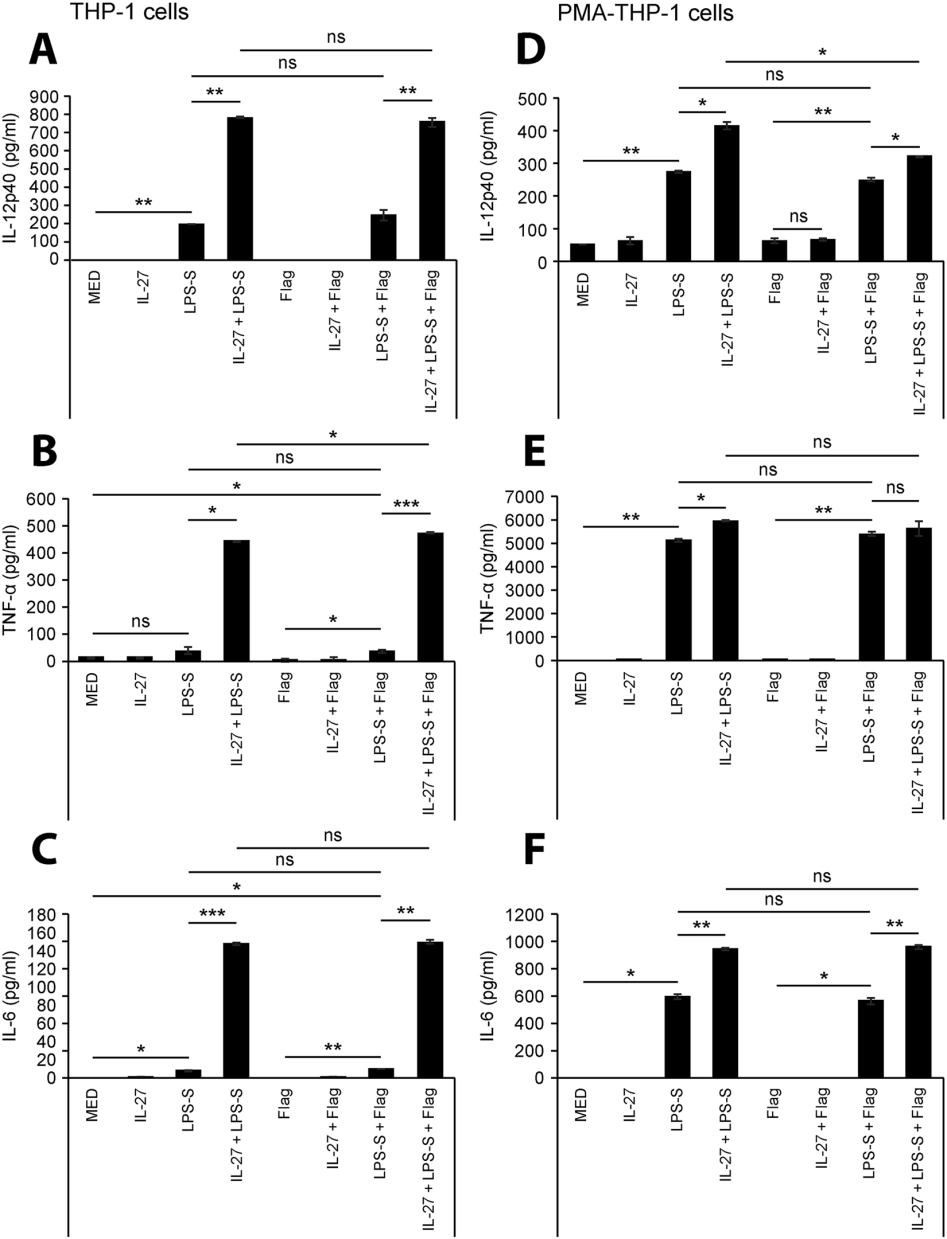
Simultaneous treatment with IL-27 results in elevated cytokine production in response to TLR4/TLR5 agonists. THP-1 cells (**A–C**) and PMA-THP-1 cells (**D–F**) were stimulated with LPS-S (100 ng/ml), flagellin (Flag) (500 ng/ml), IL-27 (50 ng/ml), or combinations of TLR agonists plus IL-27 concomitantly as indicated for 24 hours. IL-12p40 (*top panels*), TNF-α (*middle panels*), and IL-6 (*bottom panels*) levels were measured in cell-free supernatants by ELISA. Data are representative of at least three independent replicate experiments showing mean and standard deviation of ELISA technical replicates from one experiment. Mann Whitney U tests were used for statistical analyses between pairs as indicated. ns = not significant; *p≤0.05; **p≤0.01; ***p≤0.001.

Having established that compared to LPS alone, IL-27 induced a greater fold increase in NF-κB/AP-1 activity in THP-1 cells when co-stimulated with LPS-S than LPS-E, and IL-27 enhanced TLR4 and TLR5 expression in THP-1 and PMA-THP-1 cells, we next focused on *S. typhimurium* driven cytokine production. THP-1 and PMA-THP-1 cells were stimulated with LPS-S (100 ng/ml), *S. typhimurium* flagellin (500 ng/ml), or both in the presence or absence of IL-27 (50 ng/ml) for 24 hours. Cell-free supernatants were measured for production of IL-12p40, TNF-α, and IL-6 by ELISA. Treatment with IL-27 alone did not induce significant cytokine levels in either cell type relative to untreated controls (Fig. 4A–F). In line with NF-κB/AP-1 activation observed in Fig. 2, IL-12p40, TNF-α, and IL-6 production were significantly upregulated in THP-1 cells co-stimulated with LPS-S and IL-27 for 24 hours compared to LPS-S alone (Fig. 4A–C). IL-27 treatment of PMA-THP-1 cells resulted in a moderate but significant increase in LPS-induced cytokine expression (Fig. 4D–F), but the differences compared to LPS alone were less in PMA-THP-1 relative to THP-1 cells. Notably, PMA-THP-1 cells exhibited higher levels of TNF-α and IL-6 production compared to THP-1 cells. Stimulation with flagellin did not elicit detectable cytokine production neither with nor without IL-27 in either cell type except IL-12p40 production in PMA-THP-1 cells (Fig. 4D). It is interesting to note that in both THP-1 and PMA-THP-1 cells, co-stimulation of flagellin with LPS-S did not enhance cytokine production in comparison to LPS-S alone (Fig. 4). In addition, IL-27 did not alter flagellin-mediated cytokine production in THP-1 or PMA-THP-1 cells, despite increases in TLR5 expression (Fig. 3).

### IL-27 pre-treatment enhances cytokine responses to *S. typhimurium* infection

**Figure 5:**
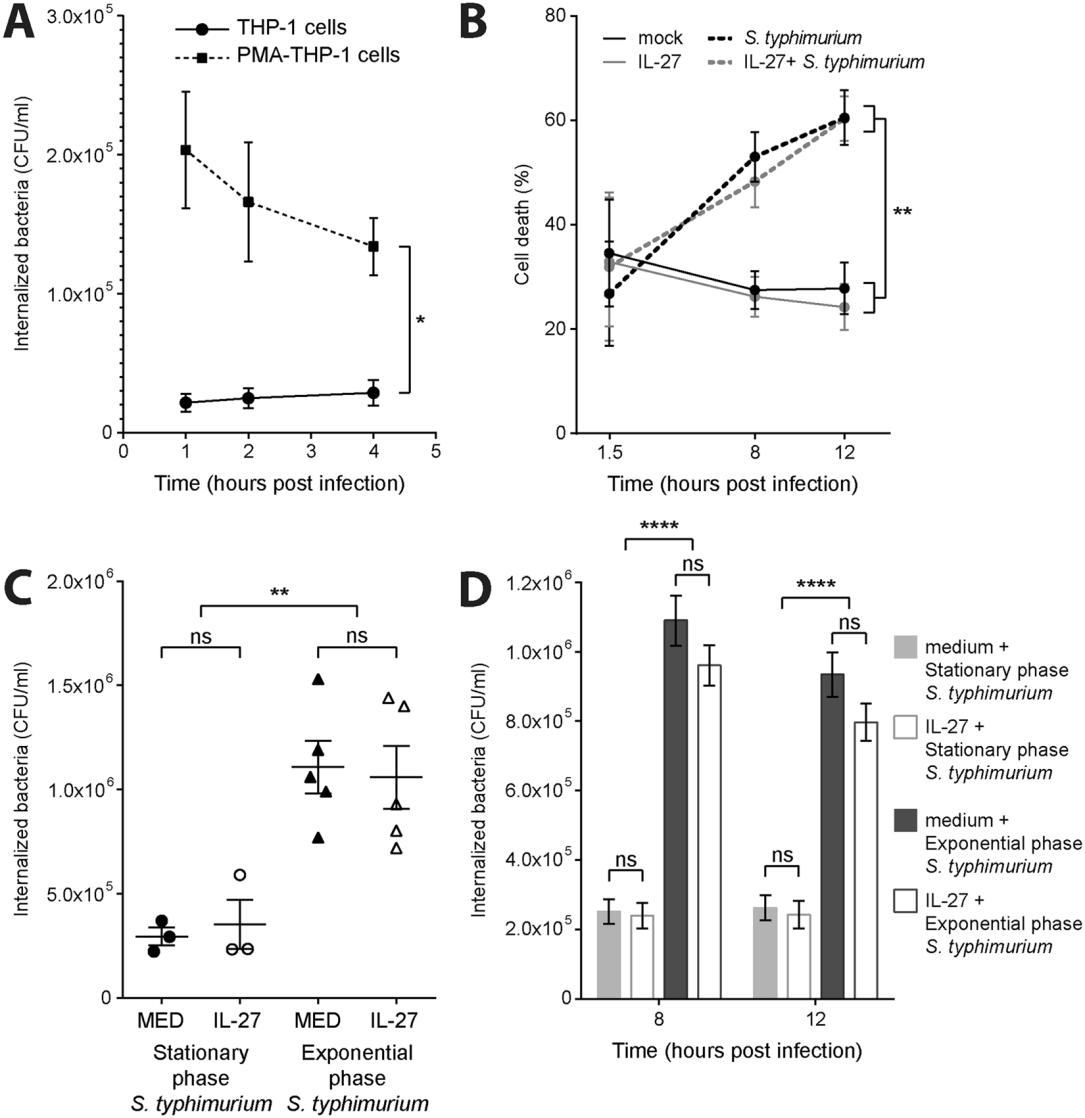
IL-27 pre-treatment does not affect *S. typhimurium* internalization or infection-induced cell death. (**A**) THP-1 cells and PMA-THP-1 cells were infected with *S. typhimurium* (MOI: 10) for 1, 2, and 4 hours. Internalized bacteria were quantified (colony forming units (CFU)/ml) using the gentamicin protection assay. (**B**) PMA-THP-1 cells were treated with or without IL-27 (50 ng/ml) for 16 hours followed by infection with stationary phase *S. typhimurium* for 1.5, 8, and 12 hours. Propidium iodide staining was used to quantify cell death of PMA-THP-1 cells by flow cytometry. (**C**) PMA-THP-1 cells were treated with or without IL-27 (50 ng/ml) for 16 hours followed by infection with stationary phase or exponential phase *S. typhimurium* for 60 or 30 min, respectively. Gentamicin protection assay was used to determine the number of internalized bacteria (CFU/ml) immediately following infection. (**D**) PMA-THP-1 cells were treated with or without IL-27 (50 ng/ml) for 16 hours, then infected with stationary phase or exponential phase *S. typhimurium* for 60 or 30 min, respectively, followed by 8 and 12 hours of incubation. PMA-THP-1 associated bacteria were quantified (CFU/ml). Mann Whitney U tests were used for statistical analyses between individual pairs as indicated. One-way ANOVA tests were used for statistical analyses for comparisons between phases of *S. typhimurium* infection in cells treated with or without IL-27 as indicated. ns = not significant; *p≤0.05; **p≤0.01; ***p≤0.001; ****p≤0.0001.

We reasoned that IL-27 treatment may modulate responses to bacterial infection. For these experiments we focused on PMA-THP-1 cells, which are more readily infected by *S. typhimurium* compared to THP-1 cells (Fig. 5A)^31^. Given that *S. typhimurium* infection induces apoptosis of PMA-THP-1 cells^32^, we tested if IL-27 could exert a protective effect. Cell death was monitored in PMA-THP-1 cells that were either cultured in medium (control) or pre-treated with IL-27 followed by stationary phase *S. typhimurium* (or mock) infection for 1.5, 8, or 12 hours. The presence of *S. typhimurium* resulted in increased PMA-THP-1 cell death from approximately 25% cell death to approximately 60% after 12 hours, as expected^32^, however, IL-27 pre-treatment did not alter cell death levels (Fig. 5B). To determine if IL-27 pre-treatment affected the ability of *S. typhimurium* to infect the cells, a gentamicin protection assay was performed on cells pre-treated with IL-27 followed by infection with either exponential or stationary phase bacteria (Fig. 5C). Exponential phase *S. typhimurium* are actively secreting virulence proteins known to enhance internalization, while stationary phase bacteria are less invasive and uptake is more dependent upon phagocytosis^33^. Higher levels of internalization of exponential phase *S. typhimurium* were detected at 1 hour post-infection compared to stationary phase at 1.5 hours post-infection; however, IL-27 did not impact the internalization of either exponential or stationary phase *S. typhimurium* (Fig. 5C). We further analyzed the impact of IL-27 on *S. typhimurium* internalization at 8 and 12 hours post infection and observed that IL-27 treatment resulted in a modest decrease in the survival of exponential phase *S. typhimurium* at these later time points, though this reduction did not reach statistical significance (Fig. 5D).

**Figure 6:**
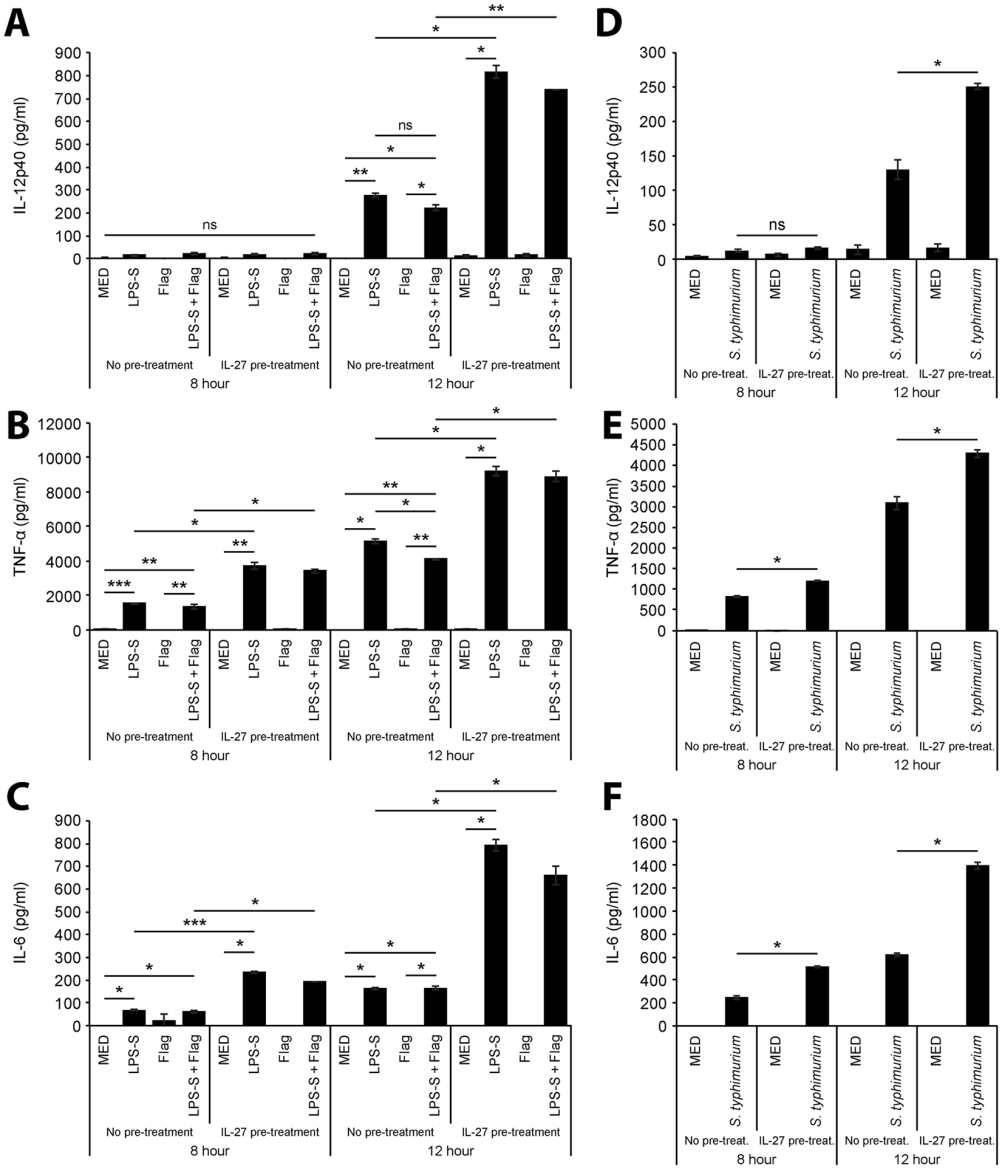
Pre-treatment with IL-27 results in elevated cytokine production in response to *S. typhimurium* components and infection. PMA-THP-1 cells were treated with or without IL-27 (50 ng/ml) for 16 hours followed by LPS-S (100 ng/ml), flagellin (Flag) (500 ng/ml), or combinations of TLR agonists as indicated (**A**–**C**) or stationary phase *S. typhimurium* (MOI: 10) (**D**–**F**) for 8 or 12 hours. IL-12p40 (*top panels*), TNF-α (*middle panels*), and IL-6 (*bottom panels*) levels were measured in cell-free supernatants by ELISA. Data are representative of at least three independent replicate experiments showing mean and standard deviation of ELISA technical replicates from one experiment. Mann Whitney U tests were used for statistical analyses between pairs as indicated. ns = not significant; *p≤0.05; **p≤0.01; ***p≤0.001.

Given that IL-27 upregulated TLR4 and TLR5 expression as well as amplified cytokine responses when co-stimulated with bacterial components, we tested if IL-27 could also enhance macrophage responses to *S. typhimurium* infection. Initially, to determine if IL-27-induced TLR4 and TLR5 expression corresponded with enhanced cytokine production, PMA-THP-1 macrophages were pre-treated with IL-27 for 16 hours prior to stimulation with LPS-S, flagellin, or LPS-S plus flagellin for 8 or 12 hours. Pre-treatment with IL-27 resulted in significantly enhanced IL-12p40 production in response to LPS-S after 12 hours compared to untreated cells (Fig. 6A), whereas IL-27 treatment increased TNF-α and IL-6 production at both 8 and 12 hours following LPS-S stimulation (Fig. 6B,C). Flagellin stimulation did not induce detectable cytokine production alone and simultaneous addition of LPS-S with flagellin did not result in enhanced cytokine production compared to LPS-S alone in either untreated or IL-27 pre-treated cells (Fig. 6A–C). To examine if IL-27 pre-treatment also could enhance macrophage responses to whole bacterium, PMA-THP-1 cells were pre-treated with IL-27 prior to infection with stationary phase *S. typhimurium* for 8 and 12 hours. Supernatants were assayed for IL-12p40, TNF-α, and IL-6 production by ELISA. Similar to stimulations using *S. typhimurium* components, IL-27 pre-treatment resulted in enhanced cytokine induction in *S. typhimurium* infected cells greater than that observed in untreated, infected cells (Fig. 6D–F). Taken together, these data indicate that IL-27 functions to amplify cytokine release triggered by *S. typhimurium* infection in human macrophages.

## Discussion

Our data show that IL-27 enhances TLR4 and TLR5 expression in human monocytes and macrophages. IL-27 treatment also enhances both TLR4- and TLR5-mediated NF-κB/AP-1 activation in human monocytes, but not in macrophages. Furthermore, co-stimulation with IL-27 and LPS enhances LPS-mediated cytokine production in both monocytes and macrophages, and pre-treatment with IL-27 enhances *S. typhimurium*-induced proinflammatory cytokine production. Given that *S. enteritidis* infection of murine BMDM induces IL-27 expression in addition to other cytokines^34^, our study suggests that during bacterial infection, autocrine effects of IL-27 may result in the propagation of inflammatory responses from monocytes and macrophages. This is in contrast with studies outlining the regulation of proinflammatory cytokine expression by IL-27 in the context of microbial infection that focus on *Mycobacterium tuberculosis* infection, where neutralization of IL-27 enhanced production of proinflammatory cytokines such as TNF-α and IFN-γ as well as anti-microbial activity^35,36^. However, WSX-1-deficient mice are more susceptible to *Listeria* infection and show impaired initiation of Th1 responses^37,38^, while the addition of IL-27 to neutrophils *in vitro* resulted in significantly enhanced IL-1β and TNF-α production and bacterial survival following *Burkholderia pseudomallei* infection^39^. Collectively, these studies demonstrate a role for IL-27 in modulating inflammatory responses that is dependent on the type of bacterial infection model.

It is important to note that TLR4 has an essential function in controlling growth of *S. typhimurium*, highlighted by the finding that TLR4 knockout mice have greater bacterial loads and significantly less proinflammatory cytokine production^40^. As well, TLR5 knockout mice or cells deficient in TLR5, also exhibit reduced cytokine production in response to flagellin or *S. typhimurium* infection^41–43^. Furthermore, TLR4 and TLR5 are thought to cooperate for optimal anti-bacterial responses^41,44^. Therefore, IL-27-mediated modulation of these receptors may enhance cellular responses to *S. typhimurium* infection.

A growing body of evidence suggests a link between IL-27 and LPS-responsiveness in myeloid cells. Firstly, Kalliolias and Ivashkiv discovered that human monocytes in the presence of macrophage colony stimulating factor (M-CSF) treated with IL-27 and LPS had enhanced TNF-α and IL-6 mRNA and cytokine production in a STAT1 dependent manner relative to without IL-27 treatment^8^. Similarly, eosinophils co-stimulated with IL-27 and LPS induced greater IL-8 production compared to LPS alone^45^. In turn, our group demonstrated that IL-27 pre-treatment of primary human monocytes enhanced LPS-induced IL-6, TNF-α, MIP-1α, and MIP-1β expression^11^. IL-27 pre-treatment amplified LPS-induced NF-κBp50 and p65 phosphorylation as well as induction of TLR4 expression and greater TLR4-CD14 colocalization^11^. We also showed that concurrent exposure of monocytes to IL-27 and LPS resulted in enhanced LPS-induced inflammasome formation and IL-1β production^13^. Interestingly, IL-27 enhanced both the LPS receptor, TLR4, as well as the purinergic ATP receptor, P2X7, for greater inflammasome activation^13^. Together, these studies support the notion that IL-27 amplifies LPS-induced cytokine induction in myeloid cells.

In this study, we demonstrated increased TLR5 expression in response to IL-27 stimulation, indicating a previously undefined role for IL-27 in the regulation of TLR5-dependent anti-bacterial responses. Moreover, we observed enhanced flagellin-induced NF-κB/AP-1 activation in THP-1 XBlue cells in the presence of IL-27, demonstrating that IL-27 is also capable of enhancing TLR5-mediated signaling in monocytes. However, we were unable to detect differences in cytokine expression in response to flagellin treatment of THP-1 or PMA-treated THP-1 cells. Others have detected IL-6 and IL-8 expression in THP-1 cells in response to flagellin proteins derived from *Treponema pallidum* at concentrations of 1–10 μg/ml^46^. In agreement with our data, *S. typhimurium* flagellin at 100 ng/ml did not induce TNF-α, IL-12p40, or IL-1β production in THP-1 cells, although TNF-α mRNA induction was detected^47^. Treatment of THP-1 cells with 2.5–100 ng/ml of *Salmonella* flagellin induced IFN-γ-inducible protein 10 (IP-10) and IL-8 production^48,49^; however, we did not examine these chemokines in our model. It is possible that in our model, the dose of 500 ng/ml of flagellin was insufficient to induce detectable levels of IL-12p40, IL-6, or TNF-α secretion despite significant NF-κBp65 phosphorylation and NF-κB/AP-1 activation.

Upon infection, monocytes are recruited to tissue to differentiate into macrophages or dendritic cells^50^. Prior to differentiation, proinflammatory monocytes are categorized by high CD14 and low CD16 expression, whereas patrolling monocytes tend to have lower CD14 and higher CD16 expression^50,51^. Proinflammatory monocytes induce phagocytosis and therefore have greater anti-microbial capacity through secretion of proinflammatory cytokines^52,53^. In this study, we used THP-1 cells as a model for human monocytes and macrophages as they respond similarly to LPS^54–56^. PMA-treatment of THP-1 cells enhances surface CD14 expression and differentiates the cells into a macrophage phenotype^57^. PMA-differentiation of human promonocytic U38 cells exhibit enhanced TNF-α production in response to *Salmonella* flagellin compared to non-PMA treated U38 cells^58^. In agreement with this, we observed greater cytokine production in PMA-THP-1 cells compared to THP-1 cells in response to LPS derived from both *E. coli* and *S. typhimurium* as a result of the differentiation state of the cells. Moreover, IL-27 treatment further enhanced cytokine production in *S. typhimurium* infected PMA-THP-1 cells. Heightened cytokine induction by human macrophages could aid in clearance of bacterial infection by recruiting adaptive immune cells. Indeed, IL-27 has been linked to promoting CD4 T helper cell-mediated protection against bacterial infection^59,60^.

Interestingly, in THP-1 cells, the phosphorylation levels of NF-κBp65 did not directly correlate with transcriptional activity levels detected by the SEAP assay in cells simultaneously treated with IL-27 and LPS or flagellin. In particular, IL-27 addition resulted in decreased LPS/flagellin-induced NF-κBp65 phosphorylation in THP-1 cells, while the SEAP assay demonstrated that IL-27 enhanced NF-κB/AP-1 activity. The potential negative regulatory effects of co-stimulation with IL-27 on NF-κBp65 phosphorylation in response to bacterial components in THP-1 cells may suggest crosstalk between IL-27- and TLR-mediated signaling pathways. Conversely, phosphorylation of NF-κBp65 in PMA-THP-1 cells correlated with the NF-κB/AP-1 activity detected by the SEAP assay. Overall, the observed differences are likely due to the different read-outs of these assays, with the SEAP assay providing information on combined AP-1 and NF-κB transcriptional activity. The absence of significant differences in TLR4- and TLR5-induced signaling activity between PMA-THP-1 cells treated with IL-27 plus bacterial components (LPS and flagellin) compared to the components alone suggests that transcription factors other than NF-κB or AP-1 are responsible for the greater induction of cytokine expression in IL-27-treated cells. Furthermore, it is possible that IL-27 may impact other molecules involved in processes such as mRNA stability, protein transport, or cytokine secretion.

Gram-negative bacterial infection of myeloid cells is well-described to induce IL-27 production^34,60,61^; this study describes IL-27 as a key player in enhancing proinflammatory response to infection with *S. typhimurium* or its components. Stimulation of THP-1 cells or PMA-THP-1 cells with IL-27 increases TLR4 and TLR5 expression on the cell surface, inducing greater LPS/flagellin-mediated signaling and downstream IL-12p40, TNF-α, and IL-6 production. Although IL-27 does not affect *S. typhimurium* internalization, it does enhance cytokine production for a more robust immune response to fight and clear bacterial infection. Elevated inflammatory responses upon IL-27 treatment prior to Gram-negative bacterial infection could potentiate greater clearance of infection due to induction of adaptive immune responses.

## Materials and Methods

### Cell lines, Cell Culture, and Reagents

THP-1 cells were purchased from the American Type Culture Collection (ATCC, Manassas, VA) and were cultured in RPMI 1640 medium (Gibco by Life Technologies, Carlsbad, CA) supplemented with 10% fetal bovine serum (FBS) (Hyclone, Logan, UT). THP-1 XBlue cells were purchased from InvivoGen (San Diego, CA). Zeocin (200 μg/ml; InvivoGen) selection antibiotic was added to Zeocin-resistant THP-1 XBlue cells in culture. THP-1 and THP-1 XBlue cells were differentiated into macrophage-like cells by culturing cells in RPMI medium +10% FBS supplemented with phorbol 12-myristate 13-acetate (PMA, 10 ng/ml; BioShop Canada Inc., Burlington, ON) for 48 hours, followed by a wash with RPMI + 10% FBS and subsequent 48 hour incubation in fresh culture medium. Recombinant human IL-27 was purchased from R&D Systems (Minneapolis, MN). LPS from *Escherichia coli* 0111:B4 (LPS-E) and LPS from *Salmonella enterica* serotype Typhimurium (*S. typhimurium*) (LPS-S) were purchased from Sigma-Aldrich (St. Louis, MO). Flagellin (Ultrapure) from *S. typhimurium* was purchased from InvivoGen.

### Peripheral blood mononuclear cells (PBMC)

Whole blood was drawn from healthy volunteers obtained in accordance with the recommendations of Canadian Tri-Council Policy Statement: Ethical Conduct for Research Involving Humans and the Queen’s University Health Sciences and Affiliated Teaching Hospitals Research Ethics Board (HSREB). All research on human samples was performed in accordance with relevant guidelines/regulations as outlined by the Queen’s University HSREB. All subjects gave written informed consent in accordance with the Declaration of Helsinki and as per the protocol approved by the HSREB. Samples were overlaid on Lympholyte Human Cell Separation Media (Cedarlane, Burlington, ON) and processed by density centrifugation for 20 min at 800 g in a 50 ml conical tube. Buffy coat containing PBMC was isolated and washed twice with PBS + 10 mM EDTA + 2% FBS and then PBMC were resuspended in RPMI + 10% FBS at 1 × 10^6^ cells/ml.

### Primary human monocyte isolation

Whole blood samples were processed with RosetteSep Human Monocyte Enrichment Cocktail (STEMCELL Technologies, Vancouver, BC), according to the manufacturer’s instructions. Processed samples were overlaid on Lympholyte-H (Human) Cell Separation Media (Cedarlane, Burlington, ON) and processed by density centrifugation for 20 minutes at 800 g in 50 ml SepMate tubes (STEMCELL Technologies). Buffy coat containing enriched monocytes was isolated and washed twice with PBS + 10 mM EDTA + 2% FBS and then monocytes were resuspended in RPMI + 10% FBS at 1 × 10^6^ cells/ml. Monocyte populations were >95% pure, as determined by CD14 staining by flow cytometry.

### Bacterial strain and culture conditions

The *Salmonella enterica* serovar Typhimurium (*S. typhimurium*) strain SL1344 was used in this study. *S. typhimurium* was streaked from frozen stock for single colonies onto LB agar plates and grown overnight at 37 °C. For stationary phase cells, a single colony was used to inoculate 3 ml of pre-warmed LB broth and grown on a rotary shaker for 16 hours at 37 °C. For exponential phase cells, 0.75 ml of a 16 hour culture was added to 24.25 ml pre-warmed LB-Miller media and cells were grown for 3.5 hours at 37 °C on a rotary shaker (200 rpm), as previously described^33^. Once the appropriate growth phase was reached, *S. typhimurium* cultures were diluted into PBS to 1 × 10^8^ cells/ml and used to infect PMA-THP-1 cells at an multiplicity of infection (MOI) of 10 for 30 minutes (exponential phase) or 60 minutes (stationary phase) at 37 °C in 5% CO_2_.

### Gentamicin protection assay

After infection with bacteria, gentamicin (20 μg/ml; BioShop Canada Inc.) was added to the mixed cultures for 30 minutes in order to kill extracellular *S. typhimurium*. Culture supernatants were then removed and cells were lysed in 0.1% v/v Triton X-100 + 0.01% w/v SDS in PBS. Cell lysates were serially diluted in PBS and plated onto LB agar to determine the total colony forming units (CFU) of internalized *S. typhimurium*. A modified gentamicin protection assay was used to quantify the surviving internalized *S. typhimurium* over time. After infection and gentamicin treatment, culture supernatants were removed and pre-warmed RPMI 1640 + 10% FBS supplemented with 20 μg/ml gentamicin was added to each well. Cells were incubated for up to 12 hours for cytokine production analysis of supernatants as well as PMA-THP-1 cell lysis and *S. typhimurium* quantification.

### ELISA

Cytokine expression was quantified in cell-free supernatants according to manufacturer’s instructions for human IL-12p40, TNF-α, and IL-6 (Thermo Fisher Scientific Invitrogen eBioscience, Carlsbad, CA). Absorbance was measured with the ELx800 Microplate Reader (BioTek, Winooski, VT) at 450 nm. Data are representative of the average ± S.D. from at least 3 independent experiments.

### Immunoblotting

Cell pellets were lysed with lysis buffer (1 M HEPES, 0.5 M NaF, 0.5 M EGTA, 2.5 M NaCl, 1 M MgCl_2_, 10% glyercol, 1% Triton X‐100) with PhosSTOP phosphatase inhibitor (Roche, Basel, Switzerland). Protein concentrations were measured with a Bradford assay (Sigma-Aldrich). Samples (10 μg protein) were separated on a 10% polyacrylamide SDS-PAGE in Tris-HCl buffer then transferred onto polyvinylidene difluoride membranes (Bio-Rad Laboratories, Hercules, CA). Membranes were blocked in 2.5% bovine serum albumin (BSA; BioShop Canada Inc.) in Tris-buffer saline (TBS) with 0.1% Tween (BioShop Canada Inc.) (TBST) for 2 hours. Membranes were subsequently probed with anti-phospho-NF-κBp65 (1:1000) (New England Biolabs, Whitby, ON) and anti-pan-NF-κBp65 (1:300) (Santa Cruz Biotechnologies, Dallas, TX) in 2.5% BSA (BioShop Canada Inc.) and mouse anti-rabbit IgG-HRP secondary antibody (1:10,000) in 2.5% BSA (BioShop Canada Inc.). Immunoblots were visualized using Clarity ECL (Bio-Rad Laboratories) on the Alpha Innotech HD2 imager using HD2 Imaging software (Thermo Fisher Scientific) for imaging. Full membrane images are provided (Supplementary Figure 1).

### SEAP QUANTI-Blue™ assay

NF-κB/AP-1-inducible secreted embryonic alkaline phosphatase (SEAP) was measured as per the manufacturers’ instructions. Briefly, THP-1 XBlue cells were plated at 2 × 10^5^ cells/ml and stimulated with combinations of medium, IL-27, LPS-E, LPS-S, and/or flagellin for 24 hours at 37 °C with 5% CO_2_. SEAP production was quantified by combining 20 μl of cell-free supernatant with 180 μl QUANTI-Blue™ buffer (InvivoGen) and incubated at 37 °C for 4 hours. Optical density was measured at 650 nm on a Varioskan spectrophotometer (Thermo Fisher Scientific, Hampton, NH).

### Flow cytometry

For surface staining, cells were resuspended in FACS buffer (PBS + 0.01% sodium azide + 2% FBS) and incubated with anti-human TLR4 AlexaFluor488 (Thermo Fisher Scientific Invitrogen eBioscience) and anti-human TLR5 AlexaFluor647 (R&D Systems). Percentage of cell death was quantified using propidium iodide (Sigma-Aldrich). Data were acquired with the Epics XL-MCL or CytoFLEX flow cytometers (Beckman Coulter, Pasadena, CA) and analyzed using FlowJo software, version X 10.0.7r2.

### Statistical analysis

Statistical significance was determined using Mann Whitney U tests between specified pairs. In Fig. 5B–D, one-way ANOVA tests were used for comparisons between groups.

## Electronic supplementary material

Supplementary Figure 1

## Data Availability

All data generated during and/or analyzed during the current study are available from the corresponding author upon request.
