# Supplementary material for: IL-27 amplifies cytokine responses to Gram-negative bacterial products and Salmonella typhimurium infection
Source: Sci Rep. 2018 Sep 12;8:13704. doi: 10.1038/s41598-018-32007-y (PMC6135775; doi:10.1038/s41598-018-32007-y)
Supplement: Supplementary file 1 — Supplementary Figure 1 [file 41598_2018_32007_MOESM1_ESM.pdf]

IL-27 amplifies cytokine responses to Gram-negative bacterial products and *Salmonella typhimurium* infection

C. Petes*, N. Odoardi*, S. M. Plater*, N. L. Martin*, K. Gee*

***** Department of Biomedical and Molecular Sciences, Queen's University, Kingston, ON, Canada, K7L 3N6

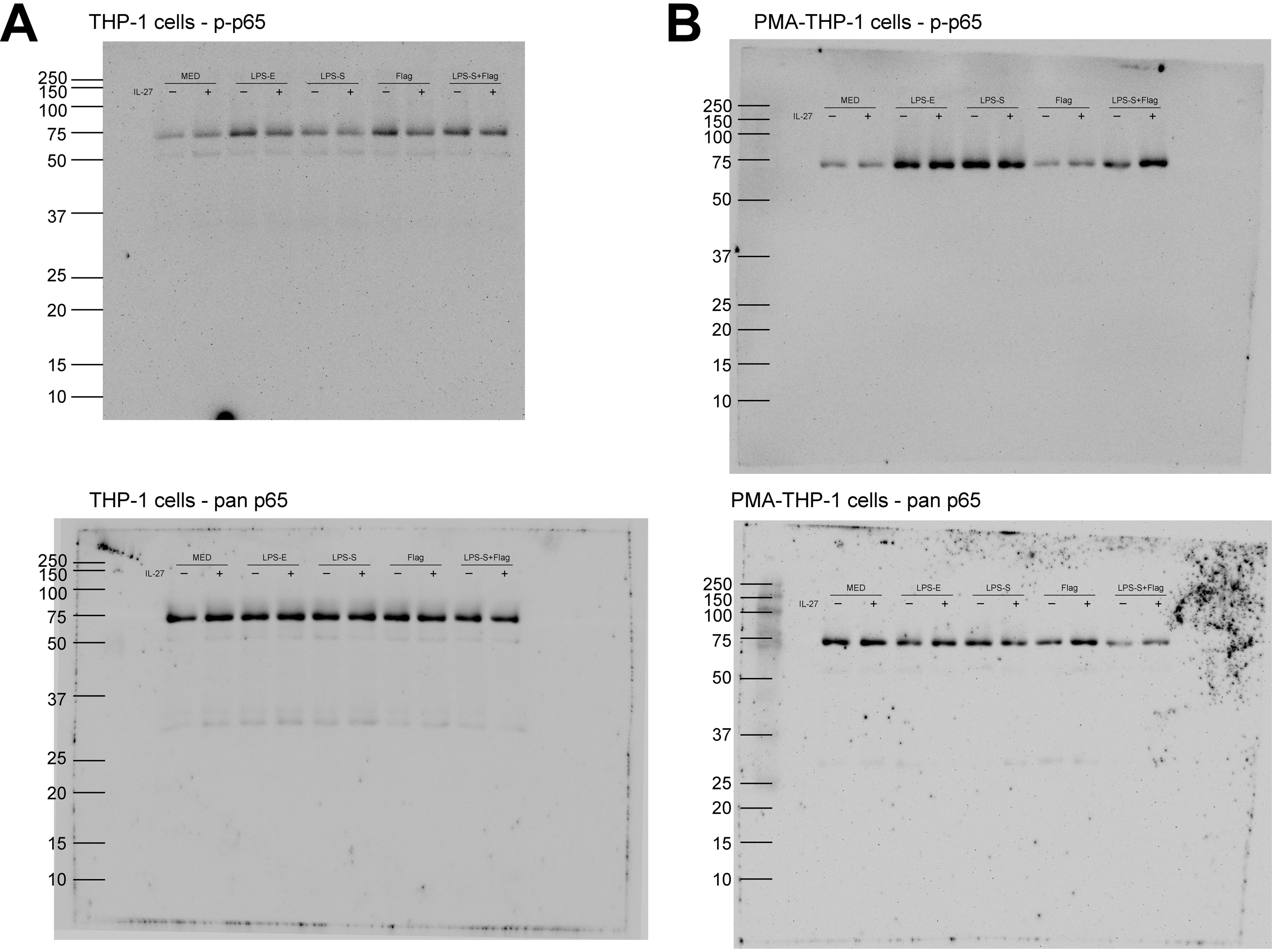

A
THP-1 cells - p-p65
250
150
100
75
50
37
25
20
15
10
MED
LPS-E
LPS-S
Flag
LPS-S+Flag
IL-27
− + − + − + − + − +
THP-1 cells - pan p65
250
150
100
75
50
37
25
20
15
10
MED
LPS-E
LPS-S
Flag
LPS-S+Flag
IL-27
− + − + − + − + − +
B
PMA-THP-1 cells - p-p65
250
150
100
75
50
37
25
20
15
10
MED
LPS-E
LPS-S
Flag
LPS-S+Flag
IL-27
− + − + − + − + − +
PMA-THP-1 cells - pan p65
250
150
100
75
50
37
25
20
15
10
MED
LPS-E
LPS-S
Flag
LPS-S+Flag
IL-27
− + − + − + − + − +

Supplementary Figure 1. Full immunoblot membranes. THP-1 cells (A) and PMA-THP-1 cells (B) were stimulated with LPS-E (100ng/ml), LPS-S (100ng/ml), flagellin (Flag) (500ng/ml), IL-27 (50ng/ml), or combinations of TLR agonists plus IL-27 concomitantly as indicated for 15 minutes. Phosphorylation of NF-κBp65 subunit (p-p65) was presented using immunoblotting on whole cell lysates. Membranes were stripped and re-probed for pan p65 as a loading control. Membranes shown are representative of three independent experiments.
